# Supplementary material for: Levels of biomarkers associated with subconcussive head hits in mixed martial arts fighters
Source: PeerJ. 2024 Aug 28;12:e17752. doi: 10.7717/peerj.17752 (PMC11365479; doi:10.7717/peerj.17752)
Supplement: Supplemental Information 2 — Notes: BDNF: Brain Derived Neurotrophic Factor; UCHL-1: ubiquitin C-terminal hydrolase; GFAP: glial fibrillary acidic protein; SD: Standard deviation; Time 01: Baseline, time 02: immediately after sparring and Time 03: 72 h after the sparring session. [file peerj-12-17752-s002.docx]

Supplementary material 01: Mean of biomarker levels for each group evaluated (n=30)

|  | **Fighters** | **Active control** | **Sedentary control** | Effect Size |
| --- | --- | --- | --- | --- |
|  | n=10 | n=10 | n=10 |  |
| Mean (SD) | | | | |
| **BDNF – Time 01^a^** | 588.5 (79.0) | 423.9 (144.3) | 514.4 (177.9) | 1.263 |
| **BDNF – Time 02** | 613.7 (81.1) | - | - | - |
| **BDNF – Time 03** | 480.0 (106.0) | - | - | - |
| **UCHL-1 – Time 01^b^** | 2778.5 (524.7) | 2782.4 (406.4) | 2734.8 (313.7) | 0.112 |
| **UCHL-1 - Time 02** | 2203.5 (881.5) | - | - |  |
| **UCHL-1 – Time 03** | 2496.5 (249.2) | - | - |  |
| **GFAP – Time 01^c^** | 3.6 (0.4) | 3.6 (0.3) | 3.6 (0.7) | 0.0000 |
| **GFAP – Time 02** | 3.6 (0.4) | - | - |  |
| **GFAP – Time 03** | 3.6 (0.5) | - | - |  |

Notes: BDNF: Brain Derived Neurotrophic Factor; UCHL-1: ubiquitin C-terminal hydrolase; GFAP: glial fibrillary acidic protein; SD: Standard deviation; Time 01: Baseline, time 02: immediately after sparring and Time 03: 72 hours after the sparring session. a. df=2; F=6.35; b. df= 2; F=6.11; c. df=2; F=6.22.

;
